# Cerebellum and micturition: what do we know? A systematic review

**DOI:** 10.1186/s40673-020-00119-9

**Published:** 2020-07-18

**Authors:** Laure Bastide, Anne-Geneviève Herbaut

**Affiliations:** Service de Neurologie, Université Libre de Bruxelles-Hôpital Erasme, Route de Lennik 808, 1070 Bruxelles, Belgium

**Keywords:** Cerebellum, Micturition, Urination, Urinary bladder, Neural control of lower urinary tract

## Abstract

**Aims:**

Micturition depends on a complex voluntary and involuntarily neuronal network located at various levels of the nervous system. The mechanism is highly dependent on the hierarchical organization of central nervous system pathways. If the role of the cortex and brainstem centres is well established, the role of other subcortical areas structures, such as the cerebellum is poorly understood. We are interested in discussing the current knowledge on the role of cerebellum in micturition.

**Methods:**

A systematic search is performed in the medical literature, using the PubMed database with the keyword « cerebellum ». The latter is combined with «urination » OR « micturition » OR « urinary bladder ».

**Results:**

Thirty-one articles were selected, focussing on micturition and describing the role of the cerebellum. They were grouped in 6 animal experimental studies, 20 functional brain imaging in micturition and 5 clinical studies.

**Conclusions:**

Although very heterogeneous, experimental and clinical data clearly indicate the cerebellum role in the micturition control. Cerebellum modulates the micturition reflex and participates to the bladder sensory-motor information processing. The cerebellum is involved in the reflex micturition modulation through direct or indirect pathways to major brainstem or forebrain centres.

## Introduction

Micturition is depending on a complex neuronal network located at various levels of the peripheral and central nervous system (CNS). It implicates autonomic and somatic systems and it is characterized by a switch-like activity pattern regulated by involuntarily and voluntary control. During the bladder filling, storage reflexes are activated and organized in the spinal cord. This involuntary spinal storage system is regulated by an involuntary brainstem voiding system. The brainstem centres are mainly composed by the pontine micturition centre (PMC) which is under the periaqueductal grey (PAG) control. In turn, the PAG is regulated by a cortical network with the prefrontal cortex (PFC) as the main final voluntary trigger. Other subcortical areas such as the cerebellum exert direct or indirect modulatory influences on the voiding reflex [1]. If the role of cortical (PFC, insula, anterior cingulate cortex [ACC], supplementary motor area [SMA]) and brainstem centres (PMC and PAG) is well established, the subcortical circuit has been less studied, particularly the role of the cerebellum.

The past twenty years have revealed the cerebellum essential role in cognition (learning and memory), emotional behaviour [2], non-somatic activities such as visceral [3, 4] and immunological responses [5]. The cerebellum contributes to generate integrated and coordinated somato-visceral responses to internal and external environmental changes. In brief, the cerebellum could be subdivided in 3 cortical parts based on their afferents: The cerebro-cerebellum located laterally in the main part of the cerebellar hemispheres which receives indirect afferences from numerous cerebral cortical areas and contributes to the regulation of precise movements. The spino-cerebellum occupies the paramedial position between the cerebellar hemispheres and the median part (also called vermis). It is the only one to receive direct afferences from the medulla. It participates in the axial, proximal and ocular motor control. At last the vestibulo-cerebellum, composed by the infero-caudal lobe (or flocculo-nodular lobe), receives afferences from the vestibular nodes and is implicated in the regulation of posture, equilibrium and in the vestibulo-ocular reflexes. Each cerebellar hemisphere contains 4 nuclei: the dentate nucleus, two interposed nuclei and the fastigial nucleus (FN). The FN is located closest to the middle line at the anterior end of the superior vermis. It seems to be involved in various non-motor structures and participates in the regulation of various visceral activities [6].

In this article, we will try to summarize the current role of the cerebellum in micturition according to experimental and clinical studies.

## Methods

**Fig. 1:**
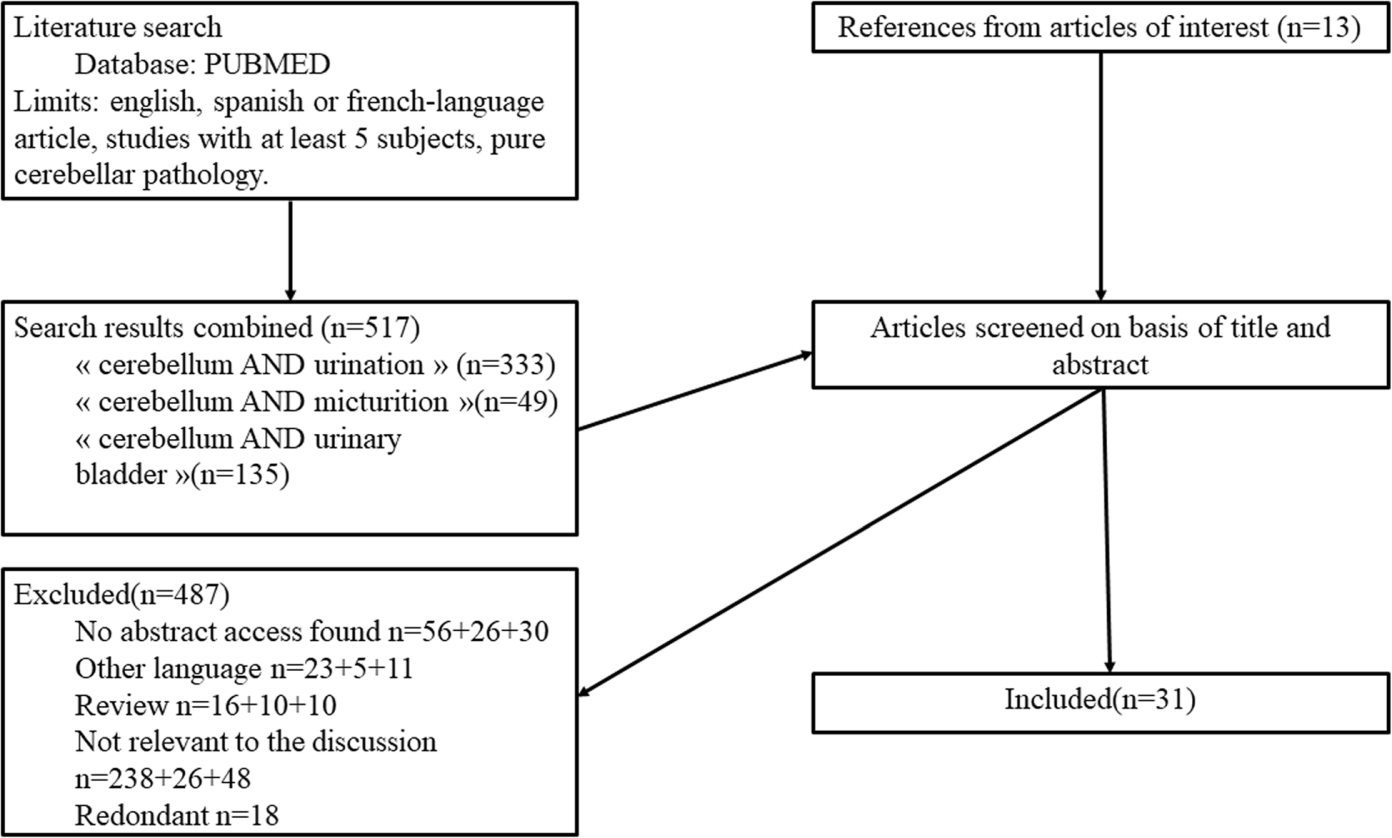
PRISMA flow diagram of studies on the cerebellum role in micturition

The search in the medical litterature was performed using the PubMed database (http://www.pubmed.org) from no limited years to April 30th 2018: the keyword « cerebellum » was combined with « urination » OR « micturition » OR « urinary bladder », bringing out respectively 333, 49, and 135 articles. At first a selection was done based on the abstract, when it was available. Then, each article was reviewed and selected according to our inclusion criteria which were: i) any experimental studies with animals or more than 5 volunteers, ii) clinical studies must have at least 5 subjects affected by pure cerebellar disorder, iii) written in English, French or Spanish language. Case reports, reviews and article without any abstract available were excluded. In addition, we added relevant articles from the reference lists of articles from the primary search. The PRISMA flow diagram is shown in Fig. 1.

## Results

Thirty-one relevant studies were included: 6 animal experiments, [7–12] 20 functional brain imaging studies [13–32] and 5 clinical studies in patients with pure cerebellar disorder [33–37]. Studies are regrouped and analysed in this order. Author’s name, year of publication, methods/aims and interesting conclusions or remarks about cerebellar implication in micturition are highlighted respectively in Tables 1, 2 and 3.

**Table 1:** Experimental animal studies

Author, year	Aim	Method	Urodynamic	Cerebellum effect
*Bradley* et al. *a et b 1969*	Modulation of reflex micturition	Electrostimulation of fastigial nucleus, pelvic and pudendal nerves in decerebrate cats and ablation of the cerebellar anterior lobe	Cystometry during filling phase	Tonic depressant effect on micturition reflex which is organized in the pontine mesencephalic reticular formation. Stimulation of pudendal and pelvic nerves afferents demonstrated a bilateral projection to the anterior and posterior vermis.
*Lisander* et al. *1974*	Modulation of bladder mobility	Electrostimulation of fastigial nucleus in cats and after infusion of guanethidin (sympatholytic drug).	Cystometry during filling phase	Inhibition of the bladder mobility induced by saline filling. Suppression of the efferent parasympathetic discharge by a possibly inhibitory action at the spinal level
*Martner* et al. *1975*	Modulation of reflex micturition	Electrostimulation of fastigial nucleus in cats with bladder outlet occluded	Cystometry during filling phase	Fastigial inhibitory influences on the spinal parasympathetic reflexes controlling bladder
*Nishizawa* et al. *1989*	Modulation of reflex micturition	Cerebellectomy in decerebrate dog	Cystometry and EMG of the urethral sphincter	Inhibitory role in the collecting phase and a facilitator role in the emptying phase.
*Deruyver* et al. *2015*	Micturition cycle and change in brain metabolism	PET study in rats	Cystometry	Hypometabolism in the rigth cerebellum during volume-induced voiding or isovolumetric bladder contraction

**Table 2:** Functional brain imaging studies

	Empty bladder	Filling phase	Voiding phase	First desire	Urge to void	Pelvic floor control
**Vermis/anterior cerebellar lobe**	None	Griffiths 2005 Sakakibara 2004 Kitta 2006	Sakakibara 2004 Dasgupta 2005		Seseke 2006 Seseke 2008	Blok 1997
**Lateral lobes**	None	Matsuura 2002 (L) Athwal 2001 (B)			Kuhtz-Buschbeck 2005 (L)	Zhang 2005 (R)
**Both area**	None	Nour 2000	Nour 2000		Blok 2006 Di Gangi Herms 2006 (L)	Seseke 2006 Seseke 2008
**Cerebellum without any precision**	None	Herzog 2008 Hruz 2007 Mehnert 2008 Pontari 2010	Shy 2014	Takao 2008		

Abbreviation: L = left; R = right; B = both

**Table 3:** Anatomo-clinical correlation studies

Author, year	Study design	Urinary symptoms	Urodynamic-EMG			
*Siffert* et al. *2000*	Retrospective case-control study of 8 patients with cerebellar mutism syndrome after a surgical lesion of midline cerebellum	UI 63%	Not available			
*Pea* et al. *2008*	Prospective, 30 patients with pure cerebellar syndrome	UI 77% (23 on 30 patients)	Overactive bladder without dyssynergia 78% (18 on 23 patients) Overactive bladder with dyssynergia 22% (5 on 23 patients) Reduced sensitivity threshold 100% PVR > 100 mL 13% (3 on 23 patients)			
*Zago* et al. *2010*	Retrospective, 75 patients affected by pure or heredodegenerative cerebellum injuries	UI 84% Frequency-nocturia 73% Dysuria 18%	Overactive bladder without dyssynergia 79% Overactive bladder with dyssynergia 3% Non-inhibited detrusor contraction 19%			
*Tateno* et al. *2012*	Prospective, 9 patients with spinocerebellar ataxia 6	UI 33% Frequency-nocturia 44% Urgenturia 22% Dysuria 11%	DO 11%	DU 22%	DSD 0%	Mild chronic denervation 63%
*Chou* et al. *2013*	Retrospective, 15 patients with cerebellar stroke	UI and emptying symptoms 40% Urgenturia 67% Dysuria 60% Urinary retention 27% Residual urine sensation 87%	DO 53%	DU 27%	DSD 40%	Non-relaxing US 47%

Abbreviations: UI = urinary incontinence; PVR = post-void residual; DO = Detrusor Overactivity; DU = Detrusor Underactivity; DSD: Detrusor- Sphincter Dyssynergia; US = Urethral Sphincter

In Bradley [7, 8] and Martner [9] studies, the cerebellum influence on the micturition reflex is studied in decerebrate cats, with a cystometry. On the one hand, Bradley et al. record neural responses in the vermis after electrical stimulation of the pelvic detrusor nerve afferent fibers and pudendal afferents from the external urethral sphincter (EUS). On the other hand, the electrical stimulation of the FN results in attenuation of the bladder reflex contraction. The ablation of the anterior lobe reduces the threshold volume and increases the reflex contractions frequency [8]. In his second study, they observe a profound depression of cellular activity in the portion of the medial rostral pons in relation to micturition reflex and in the induction of peripheral reflex when they stimulate the FN [7]. The effect of the FN stimulation is to uniformly depress the pelvic nerve discharge and the detrusor contraction.

Lisander et al. [10], also find an inhibition of the bladder contraction by the fastigial stimulation after administration of guanethidine - a sympatholytic drug -, suggesting that fastigial pathways have an inhibitory action on the parasympathetic efferents per se. In this study [10] as well as in Martner et al.’s one, [9] they obtained a suppression but also an enhancement of the micturition reflex from the very same stimulated fastigial point. The excitatory or inhibitory role of the fastigial area is depending on the prevailing bladder tone.

In 1989, Nishizawa et al. [12] analyse for the first time the cerebellar influences on the EUS and the urodynamic during the whole micturition, in decerebrate dogs before and after cerebellectomy. They observe in all subjects, the presence of a micturition reflex characterized by bladder contractions and spasmodic contractions of EUS before and after cerebellectomy. Urodynamic parameters after cerebellectomy during the filling phase are characterized by the decrease of threshold volume as demonstrated by Bradley et al. too. During the voiding phase, the cerebellectomy is followed by the decrease of the contraction pressure and voided volume. Also cerebellectomy presents no influence on the EUS during micturition reflex.

The last animal study, based on rats, analyses brain metabolism modification by fluorodeoxyglucose positron emission tomography scan ([18F] FDG-PET) during volume-induced voiding and isovolumetric bladder contractions. This study shows a hypometabolism in the right cerebellum and a hypermetabolism in the insular cortex and the ACC during the two conditions. This hypometabolism is difficult to analyse because of the limited spatial resolution of the tomography.

These animal studies altogether indicate that the cerebellum receives afferent input from the detrusor muscle and EUS (through the pudendal and pelvic nerves) and the existence of a direct pathway from the FN to the pontine area. It could also play an inhibitory role during the filling phase and a facilitator role during the voiding phase. Its inhibitory or excitatory action seems to be modulated by the underlying bladder tone.

We identified 20 functional brain imaging studies in humans, 10 with PET [13–17, 22, 23, 25, 31, 32], 9 with functional magnetic resonance imaging (fMRI) [18–21, 24, 26–29] and 1 with single-photon emission computed tomography (SPECT) [30]. The activation of cerebellar anatomical region according to the phases of the micturition cycle is reported in Table 2.

Thirteen studies are experimental; they are based on healthy volunteers and analyse brain activation pattern modulation depending on different phases of micturition and under different conditions [13, 15–22, 24–26, 29].

Mainly, we observe a cerebellum activation during the filling phase [15–18, 22, 24] which seems to be higher when the bladder filling increases [16, 18]. It is still present in the condition of “first desire to void” [25] and “urge to void” [20, 21, 26]. Matsuura et al. [17] put forward an activation of the cerebellum during bladder distension but not during cold stimulation and neither during clitoris stimulation in the Mehnert’s study [24].

Cerebellar activation is reported in the initiation of the voiding phase [15, 29] which is induced by the relaxation of the urethral sphincter and the detrusor contraction, under voluntary and autonomic controls.

We also note that the cerebellum is activated during the pelvic floor control [13, 19, 21, 26] which is involved in both voluntary and autonomic motor activities. In this situation, a stronger activation of the vermis activity during contraction than during relaxation of the pelvic floor muscles in the context of urge to void is described [21, 26]. On the one hand, the activation of the right cerebellum during repetitive pelvic floor contractions in full-bladder condition and not during emptying condition is reported [19]. On the other hand, a vermis activation is also reported in empty bladder condition [13].

Based on the data of the Table 2, we cannot highlight distinct patterns of cerebellar activation in function of the micturition phase. Furthermore, 5 studies do not report a specific area of activation.

These neuroimaging studies show that cerebellar activation during micturition concerns “more sensory phases” (filling of the bladder, urge to void, first desire to void) as well as “more coordinated motor phases” (pelvic floor control, voiding initiation).

Based on this knowledge, we will analyze the neuroanatomic basis of micturition disorder in patients with pure cerebellar disorder. Table 3 summarizes urinary symptoms and urodynamic findings in 2 prospective and 3 retrospective cohorts of cerebellar disorders.

The most important urinary symptoms are urinary incontinence (UI) (33–84%), increased frequency-nocturia (40–73%) and urgenturia (22–60%). The first cohort study, carried on patients with acute and pure midline cerebellectomy, is very close to the animal experimental conditions. Siffert et al. report 63% of UI after the surgical cerebellar lesion but there is no urodynamic data [33].

The clinical pattern reported by Zago et al. and Pea et al. consists in an overactive bladder syndrome (OAB) correlated with a detrusor overactivity (DO) on urodynamic (78 to 79% of OAB). Surprisingly they also reported 3 to 22% of detrusor-sphincter dyssynergia (DSD). This finding is not in line with animal experimental data, probably because their cohorts are very heterogenous [34, 35]. Tateno et al. reported a cohort of spinocerebellar ataxia 6 (SCA6) with also a clinical pattern of OAB. Interestingly, there are two unexpected urodynamic findings in such condition. First of all, we note DO or detrusor underactivity (DU) with the same frequency. Secondly, EUS electromyogram (EMG) shows mild chronic denervation in 5 patients mostly men. These findings suggest that this pathology is not exactly a “pure cerebellar syndrome” and could be, a minima, associated with autonomic or anterior spinal cone dysfunction [36]. In the cohort of cerebellar strokes, [37] the clinical pattern as well as the urodynamic findings are very heterogeneous. They report decreased cystometric bladder capacity, decreased detrusor voiding pressure, and smaller voided volume as Nishizawa et al. in decerebrate dogs [12].

The neuroanatomical basis of micturition disorder is also investigated by functional imaging.

Three studies deal with neurological patients, 2 with patients with Parkinson’s disease (PD) treated [23] or not [31] by deep brain stimulation of subthalamic nucleus (DBS-STN), and one with patients with multiple system atrophy (MSA) [30]. In the first study, there is no effect of the DBS-STN on cerebellum activation but their data confirm its activation during filling versus emptying bladder condition.

The second is a PET study carried on patients with PD and urine storage symptoms. The most prominent activation during DO is found in the vermis in comparison with healthy volunteers in condition of full bladder with strong desire to void [31]. It could illustrate the finding of Martner et al. [9]: activation of the FN could give an excitatory effect on micturition reflex depending on prevailing bladder tone; or it could just reflect the major recruitment of the cerebellum in patients with PD. [38]

Sakakibara et al. [30] report a decrease of the tracer activity in the bilateral upper vermis during the storage phase as compared with healthy controls. This decrease of activity is even wider in the micturition condition. This cohort is composed by 5 patients with MSA-C and 3 MSA-P with pontocerebellar atrophy. They all have urinary symptoms: nocturia in 7/8 patients, dysuria in 5/8 patients and urgency incontinent in 5/8 patients. The main urodynamic findings are DO in 6/7 patients and PVR > 100 mL in 3/7 patients. The mixed clinical pattern is characteristic of the MSA. The DO and the vermis hypoactivity could be the consequence of the cerebellar atrophy and is in line with the previous data of animal experimental [7, 9, 12].

Four studies focus on the effect of therapeutic interventions on patients with only urological problems. One study reports in women with urinary frequency and treated by tolterodine, a deactivation of the vermis [28]. A second study highlights pelvic floor muscle training with EMG-biofeedback (PFMT) in patients with stress UI; it shows a deactivation of the vermis and the left lateral lobe after the treatment. It is supporting the cerebellar involvement in pelvic floor control and coordination [27]. At last, 2 studies focus on the effect of sacral neuromodulation (SN), one on urinary retention in women with Fowler’s syndrome [32] and the other one in urge incontinent patients [14].

In the study on 12 patients with urge incontinence, newly (activated for the first time in the PET scanner) or chronically (more than 6 months) treated by a SN, cerebellum is, although differently, activated in both group of SN. During the first hours of SN, the lateral and intermediate cerebellum is more activated; in the chronic phase, the medial cerebellum shows an increase of regional cerebral blood flow. Cerebellum reactivation is also found in women with Fowler’s syndrome treated by SN [32].

Altogether these imaging studies on the neuroanatomical basis of micturition disorders support the role of the cerebellum (particularly the vermis) in sensory or motor micturition processing [23, 32]. Otherwise, they show an activation of the cerebellum and in particular the vermis in DO or OAB conditions [28, 31] but also a deactivation in the context of urinary retention [32]. Finally, different parts of the cerebellum are involved in different conditions [14].

## Discussion

The role of the cerebellum appears to be wide. It may be inhibitory for the micturition reflex during the filling phase, as illustrated by cerebellectomy in decerebrate animals and the clinical-urodynamic results in patients with cerebellar disorders. Also a facilitating role during voiding is suggested by Nishizawa et al. and neuroimaging study in Fowler’s syndrome treated by SN [12, 32].

Experimental neuroimaging studies show the implication of the cerebellum in both phases of the micturition [15–18, 22–24, 29]. It is also activated in specific conditions: “more sensory phases” as filling or distension of the bladder, [17] urge to void, [20, 21, 26] first desire to void [25] and “more coordinated motor phase” as pelvic floor control, voiding initiation [13, 15, 19, 29].

Different part of the cerebellum could be solicited with a major trend for the implication of the vermis. The analyse of cerebellar structures implicated in humans is less precise. It shows an involvement of the vermis/anterior lobe - which is included in the spino-cerebellum- and of the lateral lobes - which corresponding to cerebro-cerebellum. These structures are activated in both phases of the micturition and also in combination with supratentorial centres in micturition (as reported in all functional brain imaging studies).

In studies with anatomo-clinical correlations, the results are heterogeneous but support the fact that cerebellum has a tonic inhibitory influence over the micturition reflex, and also a facilitator influence during bladder emptying.

In their review about functional brain imaging applied to bladder control, Fowler and Griffiths do not comment on the activation of cerebellum because of lack of systematic studies [39]. But the cerebellum is obviously included in their diagram.

Several possible pathways may be described to support the hypothesis of the involvement of the cerebellum in the micturition reflex modulation.

**Fig. 2:**
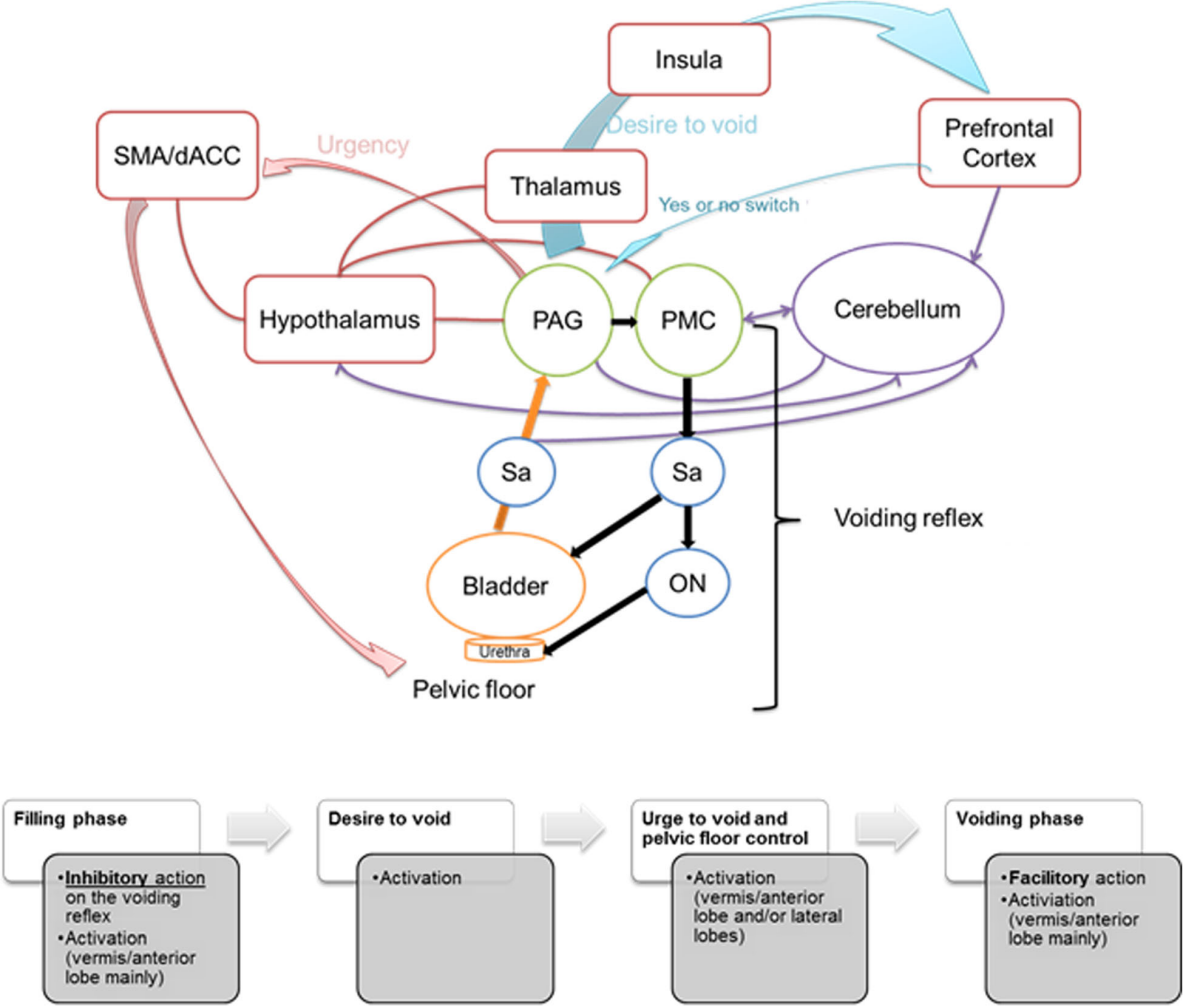
The first part is a schematic representation of the lower urinary tract control showing the voiding reflex (black arrows; Sa: sacral parasympathetic; ON: Onulf nucleus) under brainstem structures in green (PAG: periaqueductal grey; PMC pontine micturition center), forebrain structures in red (SMA: supplementary motor area; dACC: dorsal anterior cingulate cortex) involved in the perception of the desire to void circuit (blue arrows) and the urgency (red arrow). Possible, unidirectional, bidirectional connections of the cerebellum with these structures are showed by purple links and arrows. The second part (below) represents different phases of micturition with the action of the cerebellum when it is known or suggested by the animal experiments, anatomo-clinical studies or the clinical urodynamics results in patients with “pure” cerebellar disorders, and its anatomical activation when it is noticed by the experimental neuroimaging studies. (Produced with the help of the figures of references [1, 39])

As shown by animal and humans studies, the cerebellum role in micturition is mostly provided by the anterior vermis and the FN, located in the spino-cerebellum. Studies are sparse, but a direct connection with the PAG is described [40], and bidirectional neural projections are reported with the pontine micturition centre [41]. Furthermore, the FN is characterized by abundant efferents to pontine and bulbar visceral centres as well as to the reticular formation. Also numerous afferents from the medullary/pontine reticular formation, locus coeruleus, primary motor cortex and cortical motor areas on the medial wall and the hypothalamus are described [6]. Bidirectional direct connections and indirect connections mediated by autonomic areas exist between the hypothalamus and the cerebellum. The hypothalamus is well known as an important autonomic centre for regulation of visceral functions included micturition [3]. Altogether, these connections give to the cerebellum the neuroanatomical substrates to be a modulator and coordinator of the somato-visceral responses integration. A schematic representation of this information is presented in the Fig. 2.

Obviously, these findings must be interpreted with caution due to several limitations. Experimental protocols are not comparable: different species or different tools of lower urinary tract evaluation (sphincter EMG, urodynamic, electrostimulation…). In neuroimaging studies, there is no established standard protocol. Furthermore, functional brain imaging tools evaluations are different. In PET studies, the temporal resolution is limited by different isotopic markers half-life, and by the poor spatial resolution resulting from the tomography. In fMRI studies repeated trials are required to increase the signal-to-noise ratio.

In anatomo-clinical studies, the diversity of the data could be explained by the difficulties to obtain homogeneous cohorts. Pea and Zago cohorts are composed by “pure cerebellar disorder” but with a wide panel of etiologies. In the cohort of SCA 6 despite of being one of the most “pure spinocerebellar ataxia”, the urodynamic and EMG data show sacral spinal lesions. In cerebellar strokes, a mass effect of the cerebellar lesion on the brainstem could not be excluded as well as other vascular lesion of the CNS since the average age of the cohort was 75 + − 13.4 years. Nevertheless, urinary retention after cerebellar stroke is described (25% of cerebellar stroke in Umemura et al. study [42]). Cognitive disability from the Cerebellar Cognitive Affective Syndrome [2] may further interfere with micturition in these cohorts of pure cerebellar disorders. We did not include studies in other cerebellar disorders, such as FA and SCA, because of the frequent involvement of extra-cerebellar controlling micturition in these patients, which might blur the information that can be drawn regarding the cerebellar control of micturition. A last, another limitation is the number of subjects which is low in all studies.

On the clinical practice point of view, this review highlights the importance of actively tracking urinary disorders in patients with known cerebellar disorders. The implication of the cerebellum in central micturition disorders should be taken into account for further treatment research.

## Conclusion

In summary, experimental animal and human data suggest that the cerebellum and more particularly the FN, participates in a complex visceral sensory-motor program involved in the control of the micturition reflex.

Nevertheless, further clinical studies with standardized protocols combining urodynamic and fMRI evaluations are needed in patients with pure cerebellar syndrome.

## Data Availability

Not applicable.

## References

[1] de Groat WC, Griffiths D, Yoshimura N (2015). Neural control of the lower urinary tract. Compr Physiol.

[2] Hoche F, Guell X, Vangel MG, Sherman JC, Schmahmann JD (2018). The cerebellar cognitive affective/Schmahmann syndrome scale. Brain..

[3] Zhu J-N, Yung W-H, Kwok-Chong Chow B, Chan Y-S, Wang J-J (2006). The cerebellar-hypothalamic circuits: potential pathways underlying cerebellar involvement in somatic-visceral integration. Brain Res Rev.

[4] Reis DJ, Golanov EV (1997). Autonomic and vasomotor regulation. Int Rev Neurobiol.

[5] Ghoshal D, Sinha S, Sinha A, Bhattacharyya P (1998). Immunosuppressive effect of vestibulo-cerebellar lesion in rats. Neurosci Lett.

[6] Zhang X-Y, Wang J-J, Zhu J-N (2016). Cerebellar fastigial nucleus: from anatomic construction to physiological functions. Cerebellum Ataxias.

[7] Bradley WE, Teague CT (1969). Cerebellar regulation of the micturition reflex. J Urol.

[8] Bradley WE, Teague CT (1969). Cerebellar influence on the micturition reflex. Exp Neurol.

[9] Martner J (1975). Influences on the defecation and micturition reflexes by the cerebellar fastigial nucleus. Acta Physiol Scand.

[10] Lisander B, Martner J (1974). Influences on gastrointestinal and bladder motility by the fastigial nucleus. Acta Physiol Scand.

[11] Y. Deruyver, R. Rietjens, J. Franken, S. Pinto, A. Van Santvoort, C. Casteels, T. Voets, D. De Ridder, (18F)FDG-PET brain imaging during the micturition cycle in rats detects regions involved in bladder afferent signalling, EJNMMI Res. 5 (2015) 55. doi:10.1186/s13550-015-0132-0. 10.1186/s13550-015-0132-0 PMC4605920 26467154

[12] Nishizawa O, Ebina K, Sugaya K, Noto H, Satoh K, Kohama T, Harada T, Tsuchida S (1989). Effect of cerebellectomy on reflex micturition in the decerebrate dog as determined by urodynamic evaluation. Urol Int.

[13] Blok BF, Sturms LM, Holstege G (1997). A PET study on cortical and subcortical control of pelvic floor musculature in women. J Comp Neurol.

[14] Blok BFM, Groen J, Bosch JLHR, Veltman DJ, Lammertsma AA (2006). Different brain effects during chronic and acute sacral neuromodulation in urge incontinent patients with implanted neurostimulators. BJU Int.

[15] Nour S, Svarer C, Kristensen JK, Paulson OB, Law I (2000). Cerebral activation during micturition in normal men. Brain..

[16] Athwal BS, Berkley KJ, Hussain I, Brennan A, Craggs M, Sakakibara R, Frackowiak RS, Fowler CJ (2001). Brain responses to changes in bladder volume and urge to void in healthy men. Brain..

[17] Matsuura S, Kakizaki H, Mitsui T, Shiga T, Tamaki N, Koyanagi T (2002). Human brain region response to distention or cold stimulation of the bladder: a positron emission tomography study. J Urol.

[18] Griffiths D, Derbyshire S, Stenger A, Resnick N (2005). Brain control of normal and overactive bladder. J Urol.

[19] Zhang H, Reitz A, Kollias S, Summers P, Curt A, Schurch B (2005). An fMRI study of the role of suprapontine brain structures in the voluntary voiding control induced by pelvic floor contraction. Neuroimage..

[20] Kuhtz-Buschbeck JP, van der Horst C, Pott C, Wolff S, Nabavi A, Jansen O, Jünemann KP (2005). Cortical representation of the urge to void: a functional magnetic resonance imaging study. J Urol.

[21] Seseke S, Baudewig J, Kallenberg K, Ringert R-H, Seseke F, Dechent P (2006). Voluntary pelvic floor muscle control--an fMRI study. Neuroimage..

[22] Hruz P, Lövblad KO, Nirkko AC, Thoeny H, El-Koussy M, Danuser H (2008). Identification of brain structures involved in micturition with functional magnetic resonance imaging (fMRI). J Neuroradiol.

[23] Herzog J, Weiss PH, Assmus A, Wefer B, Seif C, Braun PM, Pinsker MO, Herzog H, Volkmann J, Deuschl G, Fink GR (2008). Improved sensory gating of urinary bladder afferents in Parkinson’s disease following subthalamic stimulation. Brain..

[24] Mehnert U, Boy S, Svensson J, Michels L, Reitz A, Candia V, Kleiser R, Kollias S, Schurch B (2008). Brain activation in response to bladder filling and simultaneous stimulation of the dorsal clitoral nerve--an fMRI study in healthy women. Neuroimage..

[25] Takao T, Tsujimura A, Miyagawa Y, Kiuchi H, Ueda T, Hirai T, Komori K, Takada S, Nonomura N, Osaki Y, Enomoto K, Hatazawa J, Okuyama A (2008). Brain responses during the first desire to void: a positron emission tomography study. Int J Urol.

[26] Seseke S, Baudewig J, Kallenberg K, Ringert R-H, Seseke F, Dechent P (2008). Gender differences in voluntary micturition control: an fMRI study. Neuroimage..

[27] Di Gangi Herms AMR, Veit R, Reisenauer C, Herms A, Grodd W, Enck P, Stenzl A, Birbaumer N (2006). Functional imaging of stress urinary incontinence. Neuroimage..

[28] Pontari MA, Mohamed FB, Lebovitch S, Moonat S, Lebed B, Ruggieri MR, Faro SH (2010). Central nervous system findings on functional magnetic resonance imaging in patients before and after treatment with anticholinergic medication. J Urol.

[29] Shy M, Fung S, Boone TB, Karmonik C, Fletcher SG, Khavari R (2014). Functional magnetic resonance imaging during urodynamic testing identifies brain structures initiating micturition. J Urol.

[30] Sakakibara R, Uchida Y, Uchiyama T, Yamanishi T, Hattori T (2004). Reduced cerebellar vermis activation during urinary storage and micturition in multiple system atrophy: 99mTc-labelled ECD SPECT study. Eur J Neurol.

[31] Kitta T, Kakizaki H, Furuno T, Moriya K, Tanaka H, Shiga T, Tamaki N, Yabe I, Sasaki H, Nonomura K (2006). Brain activation during detrusor overactivity in patients with Parkinson’s disease: a positron emission tomography study. J Urol.

[32] Dasgupta R, Critchley HD, Dolan RJ, Fowler CJ (2005). Changes in brain activity following sacral neuromodulation for urinary retention. J Urol.

[33] Siffert J, Poussaint TY, Goumnerova LC, Scott RM, LaValley B, Tarbell NJ, Pomeroy SL (2000). Neurological dysfunction associated with postoperative cerebellar mutism. J Neuro-Oncol.

[34] Pea U, Zago T, Areta L, Marzorati G (2008). Vesico-sphincter dysfunctions in pure cerebellar syndrome (PCS): a possible basis for the study of anatomo-functional correlations. Urologia..

[35] Zago T, Pea U, Fumagalli GL, Areta L, Marzorati G, Bianchi F (2010). Cerebellar pathology and micturitional disorders: anatomotopographic and functional correlations. Arch Ital Urol Androl.

[36] Tateno F, Sakakibara R, Sugiyama M, Kishi M, Ogawa E, Takahashi O, Yano M, Uchiyama T, Yamamoto T, Tsuyuzaki Y (2012). Lower urinary tract function in Spinocerebellar Ataxia 6. Low Urin Tract Symptoms.

[37] Chou Y-C, Jiang Y-H, Harnod T, Kuo H-C (2013). Characteristics of neurogenic voiding dysfunction in cerebellar stroke: a cross-sectional, retrospective video urodynamic study. Cerebellum..

[38] Hanakawa T, Katsumi Y, Fukuyama H, Honda M, Hayashi T, Kimura J, Shibasaki H (1999). Mechanisms underlying gait disturbance in Parkinson’s disease: a single photon emission computed tomography study. Brain..

[39] Fowler CJ, Griffiths DJ (2010). A decade of functional brain imaging applied to bladder control. Neurourol Urodyn.

[40] Dietrichs E (1983). Cerebellar cortical afferents from the periaqueductal grey in the cat. Neurosci Lett.

[41] Dietrichs E, Haines DE. Possible pathways for cerebellar modulation of autonomic responses: micturition. Scand J Urol Nephrol Suppl. 2002:16–20. 10.1080/003655902320765917 12475012

[42] Umemura T, Ohta H, Yokota A, Yarimizu S, Nishizawa S (2016). Urinary retention associated with stroke. J UOEH.

